# Large Anomalous Hall Effect, Non‐Vanishing Berry Curvature in (110) FeRh Antiferromagnet Films via Interface Strain

**DOI:** 10.1002/advs.202518999

**Published:** 2026-01-28

**Authors:** Yun‐Ho Kim, Gil‐Sung Kim, Jae Won Choi, Jung‐Min Cho, Won‐Yong Lee, Hongkee Yoon, Yeonho Choi, Shelby Fields, Steven Bennett, Mona Zebarjadi, Young‐Gui Yoon, Sang‐Kwon Lee

**Affiliations:** ^1^ Department of Physics Chung‐Ang University Seoul Republic of Korea; ^2^ Department of Semiconductor Physics Kangwon National University Chuncheon Republic of Korea; ^3^ Materials Science and Technology Division U.S. Naval Research Laboratory Washington District of Columbia USA; ^4^ Department of Electrical and Computer Engineering University of Virginia Charlottesville Virginia USA; ^5^ Department of Materials Science and Engineering University of Virginia Charlottesville Virginia USA

**Keywords:** anomalous hall effect, antiferromagnetic spin transport, berry curvature, interface strain, iron rhodium

## Abstract

The anomalous Hall effect (AHE) has been understood as a transport phenomenon usually observed in ferromagnetic and non‐collinear antiferromagnetic materials where broken time‐reversal symmetry combined with spin–orbit coupling produces a net Berry curvature. Combined spatial inversion (P) and time‐reversal (*T*) symmetries forbid an AHE in collinear antiferromagnets. In this study, we demonstrate a pronounced AHE in (110)‐oriented FeRh thin films epitaxially grown on Al_2_O_3_ substrates, even though the films remain collinear antiferromagnetic at low temperatures. Unlike the bulk B2 phase, where *PT* symmetry enforces the cancellation of Berry curvature, the epitaxial (110) orientation and substrate‐induced strain explicitly break the spatial inversion symmetry (*P*). This symmetry‐lowering mechanism, which lifts the *PT* constraint, enables a finite Berry curvature distribution in momentum space. Consequently, this allows for robust anomalous transverse transport even in the collinear antiferromagnetic regime, providing a new degree of freedom to engineer topological properties in antiferromagnets. First‐principles density‐functional calculations reproduce the strain‐induced Berry curvature and quantitatively account for the measured AHE in the 5–100 K range. Our results show that intrinsic strain can be harnessed to tailor Berry curvature in collinear antiferromagnets, opening a pathway toward antiferromagnetic spintronic applications.

## Graphical abstract

This study demonstrates a strain‐tunable anomalous Hall response in the (110)‐oriented FeRh thin film. First‐principles calculations reveal that strain‐induced symmetry lowering redistributes Berry curvature near the Fermi level via band shifts and avoided crossings. The qualitative agreement between experimental trends and theoretical predictions validates the intrinsic mechanism, offering a strategy to engineer topological transport in spintronic devices through mechanical deformation.

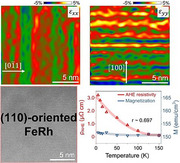

## 1 Introduction

Berry curvature induces anomalous velocity as a fictitious magnetic field in a uniform magnet in momentum space [1, 2, 3, 4]. This exhibits a monopole‐like texture around the Weyl point [5, 6, 7, 8, 9] and is widely considered to be the fundamental cause of the anomalous Hall effect (AHE). AHE, which is commonly observed in ferromagnets (FMs), can also occur in non‐collinear antiferromagnets (AFMs) under the requirement of broken time‐reversal symmetry (*T*) combined with spin–orbit coupling (SOC), which together generate a non‐vanishing net Berry curvature [3, 10, 11, 12]. In collinear AFMs, the AHE is traditionally forbidden because the presence of combined inversion (*P*) and time‐reversal (*T*) symmetry (*PT* symmetry) enforces the exact cancellation of Berry curvature throughout the Brillouin zone. Recent studies have shown that AHE can also occur in collinear AFMs due to factors such as spin canting [13] or the presence of non‐centrosymmetric atoms [14]. To induce the AHE signal in the materials, it is necessary to break specific spatial symmetries, such as inversion symmetry, to lift this *PT* constraint. This intrinsic AHE phenomenon appears in various material structures. Magnetic structures such as FMs (Co_3_Sn_2_S_2_, Fe_5_Sn_3_, Fe_3_Sn_2_, etc.) [15, 16, 17], ferromagnetic van der Waals semimetallic Fe_3_GeTe_2_ [18] spin liquids, AFMs, Weyl semimetals [19, 20, 21, 22, 23], and unconventional magnetic structures of magnetic skyrmions [24] have been reported to capture the real‐space Berry phase and induce an AHE response.

AFMs typically have net magnetization produced by an external magnetic field, and have intrinsically high dynamic frequencies because spin precession mostly occurs in the terahertz frequency range [25, 26, 27, 28]. They also have spin textures such as domain walls and skyrmions, which have attracted significant research attention recently [29, 30]. Various non‐collinear AFMs with AHE measurement platforms have been studied recently. For example, substantial AHE signals with non‐vanishing Berry curvature owing to chiral spin textures have been reported in hexagonal Mn_3_Ge [31], Mn_2_Sn [12], Mn_3_Pt [32], and Mn_3_Ir [33]. Anomalous Hall conductivity (AHC) is usually assumed not to occur in collinear AFMs because the Berry curvature becomes zero in AFMs due to symmetry constraints. However, in 2025, scanning transmission electron microscopy (STEM) experiments were conducted to observe AHE with non‐zero Berry curvature in L1_0_‐IrMn (001) thin films, although it is a collinear AFM [34]. Nevertheless, further theoretical and experimental studies on the underlying physics of non‐vanishing AHC in collinear AFMs are highly needed.

Among various AFMs, iron rhodium (FeRh), a binary equiatomic ordered alloy, is particularly suitable for novel spintronics applications [35, 36]. Bulk FeRh undergoes a magnetic phase transition at a very high temperature of 360 K, which is close to room temperature, making it very useful for spintronics device applications [37, 38]. AHE measurements of polycrystalline FeRh films have confirmed that the sign of the spin Hall conductivity in the AFM phase is opposite to that in the FM phase [38]. Studies have demonstrated the antiferromagnetic properties of single‐crystal epitaxial (100)‐FeRh films grown on MgO (100) substrates, and further, they have confirmed that in these high‐symmetry structures, the *PT* symmetry remains intact, enforcing a vanishing Berry curvature and resulting in zero AHE signals [39, 40]. Nevertheless, the theoretical and experimental understanding of the AHE and Berry curvature‐related phenomena, especially in collinear antiferromagnetic FeRh films, remains incomplete. Furthermore, an in‐depth study of the collinear AFM properties of FeRh at low temperatures is required.

Here, we report, for the first time, the AHE characteristics of FeRh thin films due to the non‐vanishing Berry curvature in collinear AFM FeRh films by combining AHE measurements in the temperature range of 5–300 K and the unique material property of the FeRh films grown in the (110) direction. These (110)‐oriented FeRh thin films were grown on Al_2_O_3_ rather than on the conventional MgO substrate. Unlike the (100) orientation, the (110) epitaxial growth on a lattice‐mismatched substrate breaks the spatial inversion symmetry due to the specific atomic stacking sequence and interface‐induced strain. This symmetry‐lowering, fundamentally distinct from the behavior of high‐symmetry (001) films, directly lifts the *PT* constraint and activates a non‐vanishing Berry curvature in the momentum space. By establishing (110)‐oriented FeRh as a symmetry‐tunable platform, we demonstrate that the observed AHE is an intrinsic consequence of the tailored topological electronic structure, rather than a parasitic effect of residual ferromagnetic moments.

## 2 Results and Discussion

### 2.1 Atomic Arrangement and Lattice Distortion in (110)‐Oriented FeRh

**FIGURE 1:**
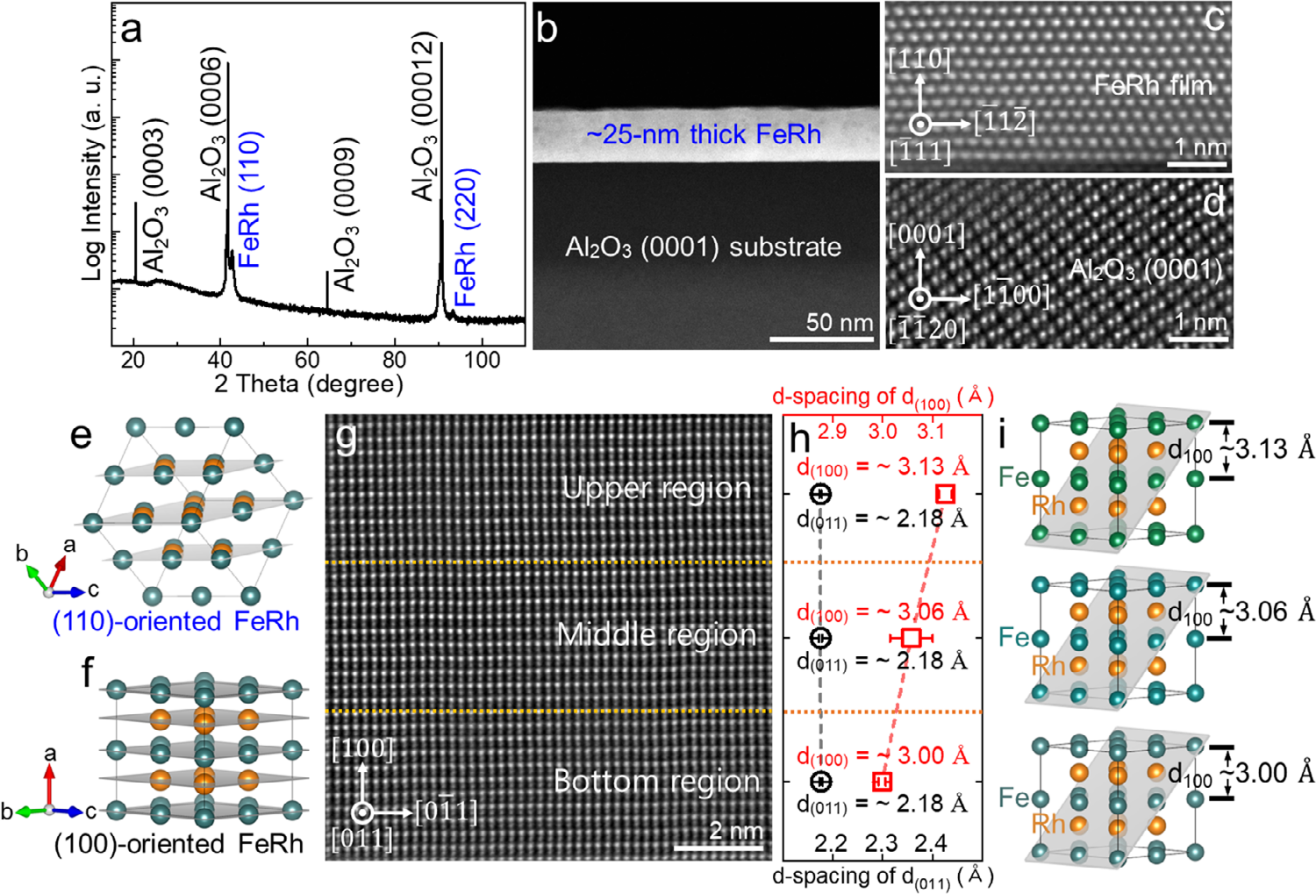
Material characteristics of the FeRh thin film on a c‐plane Al_2_O_3_(0001) substrate. (a) HR‐XRD patterns for the FeRh thin film grown on c‐plane Al_2_O_3_ (0001). (b) Cross‐sectional STEM image of the FeRh thin film. Atomic‐resolution ADF‐STEM images corresponding to the (c) FeRh and (d) Al_2_O_3_ regions, taken along the [$\bar{1}11$] zone axis of FeRh and [$\bar{1}\bar{1}20$] zone axis of Al_2_O_3_. (e,f) Atomic arrangements in the (110) and (100) planes of the [110]‐oriented and [100]‐oriented FeRh structures, respectively, were constructed using the free VESTA software. (g) ADF‐STEM image and (h) the corresponding d‐spacing values for (100) and (110) planes across the bottom, middle, and upper regions. (i) Structural configuration of the FeRh (2 × 2 × 2) supercells projected along the [011] zone axis.

FeRh thin films were grown on c‐plane Al_2_O_3_ (0001) substrates using direct‐current (DC) magnetron sputtering, and their crystallographic properties were examined using high‐resolution X‐ray diffraction (HR‐XRD) with Cu Kα_1_ radiation (Bruker D8 Discover). Figure 1a shows the θ–2θ diffraction pattern of an as‐grown FeRh film on the c‐plane Al_2_O_3_ (0001) substrate. Besides the characteristic (000*l*) peaks from the c‐plane Al_2_O_3_ substrate, two diffraction peaks at 42.63° and 93.32° were observed, corresponding to the (110) and (220) planes of the B_2_‐ordered FeRh phase (JCPDS No. 25–1408) [39, 41, 42], respectively. This confirms that the FeRh thin film grown on the c‐plane Al_2_O_3_ substrate is preferentially oriented along the [110] crystallographic direction. The structural characteristics of the (110)‐oriented FeRh thin film were further investigated using high‐resolution STEM. As shown in Figure 1b, the STEM image confirms the formation of a uniform FeRh layer with a thickness of ∼25 nm on the c‐plane Al_2_O_3_ (0001) substrate. Figure 1c,d present atomic‐resolution annual dark‐field (ADF) STEM images corresponding to the FeRh and Al_2_O_3_ regions, respectively, taken along the [$\bar{1}11$] zone axis of FeRh and [$\bar{1}\bar{1}20$] zone axis of Al_2_O_3_. These results clearly demonstrate that the (110) plane of FeRh parallels the (0001) plane of Al_2_O_3_, which is consistent with the result of the HR‐XRD observation (Figure 1a). To compare the atomic arrangements in the (110) and (100) planes of the [110]‐oriented and [100]‐oriented FeRh structures, respectively, structural models were constructed, using the free Vesta software, based on (2 × 2 × 2) supercells. As shown in Figure 1e,f, the [110]‐oriented FeRh exhibits an atomic arrangement in which non‐magnetic Rh atoms and magnetic Fe atoms coexist within (110) planes, whereas in the [100]‐oriented FeRh, the Rh and Fe atoms are spatially separated into distinct (100) planes [43]. While this coexistence is a geometric property of the B2 structure, it is important to note that in our epitaxial (110) thin films, the spatial inversion symmetry (*P*) is explicitly broken due to the specific stacking sequence and the interface environment, unlike in the ideal cubic bulk phase. Specifically, the (110) interface with the Al_2_O_3_ substrate imposes a unique stacking order that lacks a center of inversion, which is a prerequisite for a non‐vanishing Berry curvature in collinear antiferromagnets. This symmetry‐lowering is critical as it lifts the combined inversion and time‐reversal (*PT*) symmetry constraint that typically suppresses the Berry curvature in collinear antiferromagnets. To investigate the atomic distribution and crystal lattice of the [110]‐oriented FeRh, an ADF‐STEM image was acquired by tilting the specimen to lie along the [011] zone axis of FeRh. Figure 1g,h presents the ADF‐STEM image and the corresponding d‐spacing values for the (100) and (110) planes, respectively, across the bottom, middle, and upper regions. These results demonstrate the high crystallinity of FeRh thin film with a well‐defined B_2_‐ordered structure, where Fe and Rh atoms are periodically arranged in a 1:1 ratio along the (100) crystallographic direction. The d‐spacing of the (110) plane was consistently determined to be ∼2.18 Å across all regions, whereas that of the (100) plane exhibited a slight expansion from ∼3.00 Å (bottom region) to ∼3.13 Å (upper region) [44, 45]. This indicates the progressive partial strain relaxation with increasing distance from the interface and is qualitatively in agreement with the lattice strain variations obtained by geometric phase analysis (GPA, Gatan) shown in Figure S1. Figure 1i presents the resulting structural configuration of the FeRh (2 × 2 × 2) supercells projected along the [011] zone axis.

### 2.2 Magnetization Dynamics and Metamagnetic Transition in (110) FeRh Thin Films

The magnetoelastic contribution to magnetic anisotropy in FeRh thin films is strongly influenced by the strain imposed by the underlying substrate, which governs whether the spin orientation prefers an in‐plane or out‐of‐plane configuration. Notably, strain drives spin reorientation during the transition from the antiferromagnetic to the ferromagnetic phase, resulting in perpendicular magnetic anisotropy in the antiferromagnetic phase. In the ferromagnetic state, tensile strain typically favors in‐plane spin alignment, while compressive strain promotes perpendicular orientation; conversely, the opposite trend is observed in the antiferromagnetic state [46, 47, 48]. This strain‐induced anisotropy, which scales with the lattice constant ratio *c/a*, also significantly influences the magneto‐structural transition temperature [48]. These findings underscore that crystallographic orientation and substrate‐induced strain are key factors in determining the magnetic behavior of FeRh thin films.

**FIGURE 2:**
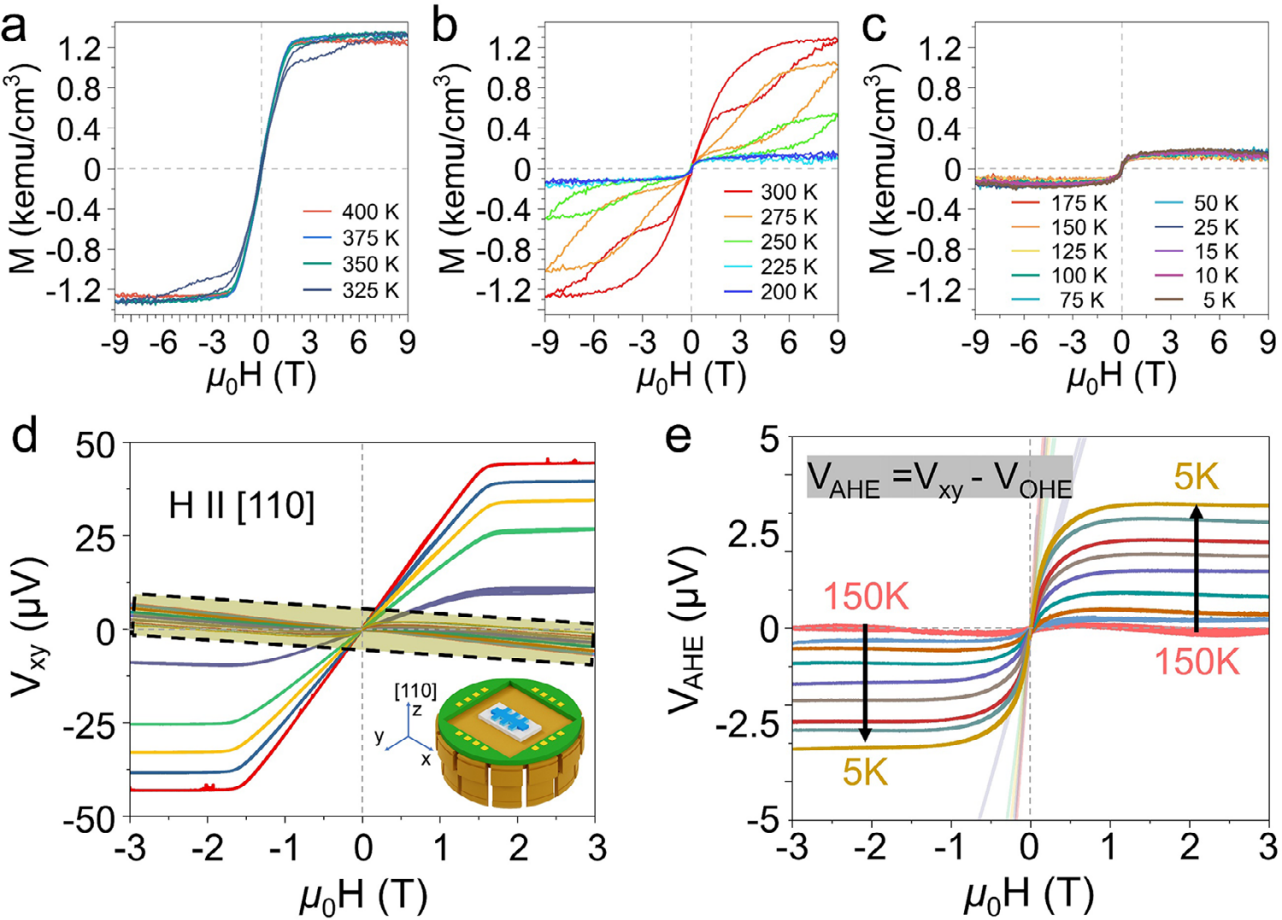
Temperature‐dependent magnetization and Hall response in (110)‐oriented FeRh thin films. (a–c) Magnetic hysteresis loops measured by VSM under out‐of‐plane magnetic fields (H ⊥ film surface) at various temperatures, showing the ferromagnetic‐to‐antiferromagnetic transition. Above 325 K (a), the sample exhibits clear ferromagnetic hysteresis. Intermediate temperatures (200–300 K; (b) reveal a gradual reduction in remanent magnetization, indicating a mixed‐phase regime. Below 175 K (c), the magnetization vanishes, confirming a transition to the antiferromagnetic phase. (d) Transverse voltage V_xy_​ measured under magnetic fields parallel to the [110] direction at various temperatures. The nonlinear behavior below 150 K indicates the presence of an anomalous Hall contribution, despite vanishing net magnetization. The inset shows the measurement configuration. (e) Extracted anomalous Hall voltage V_AHE_ = V_xy_ ​− V_OHE_​, highlighting a monotonic increase in V_AHE_ as the temperature decreases from 150 to 5 K. The growth of V_AHE_ ​ in the antiferromagnetic regime suggests a Berry curvature‐driven mechanism independent of remanent magnetization.

Building on this understanding, we next examined the temperature‐dependent magnetization of the (110)‐oriented FeRh films to explore how these structural features influence their magnetic phase evolution. We carried out detailed magnetic characterization using a vibrating sample magnetometer (VSM). Consistent with the behavior observed in the more widely studied (100)‐oriented films, the (110)‐oriented samples exhibit a sharp first‐order transition from the ferromagnetic to the antiferromagnetic phase as the temperature is lowered below room temperature (Figure 2a–c). During this transition, the magnetization traces a characteristic butterfly‐shaped hysteresis loop (Figure 2b), arising from the metamagnetic coexistence of ferromagnetic and antiferromagnetic domains and the competing anisotropies of the constituent magnetic sublattices. In the low‐temperature regime, while the antiferromagnetic state is dominant, a finite remanent magnetization of approximately 150 emu/cm^3^ remains detectable (Figure 2c). This value corresponds to roughly 10 %–15 % of the full saturation magnetization (*M*_s_ ≈ 1100 emu/cm^3^) reported for B2‐ordered FeRh. Such residual ferromagnetism is commonly observed in epitaxial FeRh thin films and can be attributed to several factors: surface/interface‐stabilized ferromagnetic regions even at nominal AFM temperatures, the kinetic arrest of FM domains during the first‐order transition, and uncompensated moments induced by local symmetry breaking or disorder in the B2 lattice [49, 50, 51, 52, 53, 54, 55, 56]. To ensure the accuracy of these measurements, complete magnetometry datasets, including sapphire substrate background subtraction and orientation‐dependent M─H curves, were carefully evaluated (Figure S2). These results confirm that the observed anomalous Hall response in the low‐temperature regime originates from an AFM‐dominant state containing a characteristic residual ferromagnetic fraction typical of high‐quality epitaxial films.

### 2.3 Anomalous Hall Effect and Berry Curvature in (110)‐Oriented FeRh Films

The observed magnetic characteristics, especially the presence of finite remanent magnetization and strain‐mediated spin reorientation, raise important questions about their influence on electronic transport phenomena. Since AHEs typically arise from magnetic states with distinct spin textures or symmetry breaking, we systematically investigated the translation of these magnetization behaviors into the anomalous Hall signals measured in the (110)‐oriented FeRh films. Accordingly, we performed Hall effect measurements in a physical property measurement system (PPMS) from 400 K down to 5 K, using 25 K steps above 25 and 5 K steps below. The FeRh film was mounted on a PPMS puck (inset of Figure 2d) so that the magnetic field lay parallel to the (110) plane; the field was swept between ±9 T while a longitudinal current (x‐direction), I_xx_​ = 5 µA, was applied. The resulting transverse (y‐direction) voltage, V_xy_, was recorded at each temperature (Figure 2d). At decreasing temperatures, we observed an anomalous Hall response below a temperature of 150 K that was distinct from the behavior at higher temperatures (highlighted by the yellow box in Figure 2d) and that grew progressively stronger down to a temperature of 5 K.

The V_xy_​ measured in magnetic field sweeps typically represents the combined contribution of both the ordinary Hall effect (OHE) and the AHE [38, 57]. In the OHE, charge carriers (electrons or holes) experience a Lorentz force perpendicular to both the I_xx​_ and magnetic field, generating a Hall voltage linearly proportional to the external magnetic field: V_OHE_ = *µ*_0_H*R*_H_ ​, where *µ*_0_​ is the vacuum permeability, and *R*_H_​ is the Hall coefficient. In contrast, the AHE arises intrinsically in magnetically ordered materials. To ensure reliable extraction of the anomalous component, the measured V_xy_ was antisymmetrized with respect to the magnetic field to eliminate longitudinal resistivity mixing, and the linear V_OHE_ was subtracted based on the high‐field slope.

Consequently, the remaining transverse voltage contains only the anomalous Hall component V_AHE_ (Figure 2e). At 150 K, the V_AHE_ is nearly zero, but it rises steadily as the temperature decreases, reaching about 3 µV at 5 K. While a finite Hall signal is also present in the ferromagnetic phase above 150 K, its magnitude is relatively small as the high symmetry of the FM state suppresses the intrinsic Berry curvature contribution. This behavior contrasts sharply with that observed in the FeRh thin film grown on a MgO substrate (see Figure S3).

**FIGURE 3:**
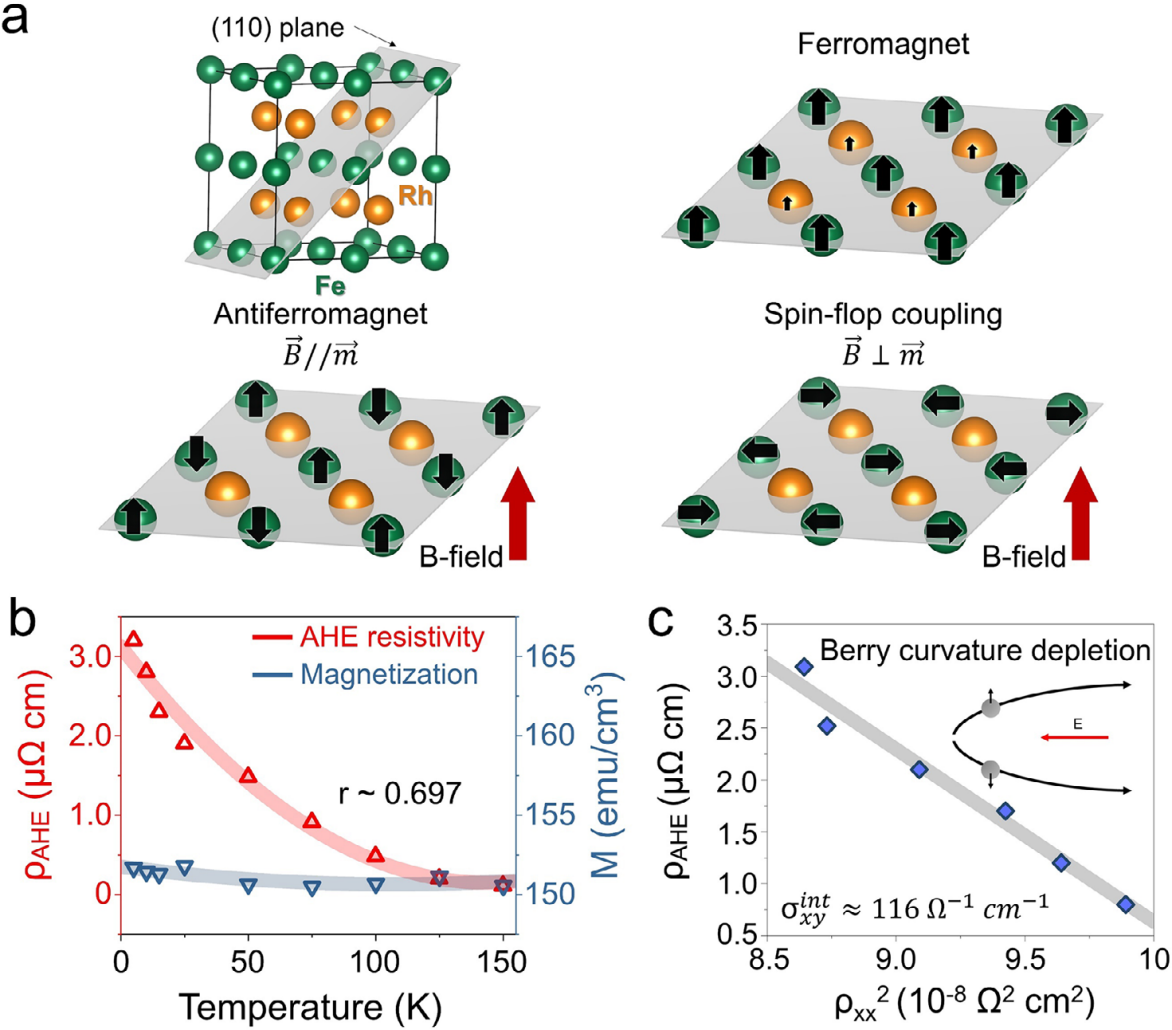
Evidence for Berry curvature‐driven anomalous Hall effect in (110)‐oriented FeRh thin films. (a) Schematic illustrations of spin configurations in the (110) plane of FeRh. Fe atoms (green) carry local magnetic moments, whereas Rh atoms (orange) are drawn without arrows in the AFM (bottom left) and spin‐flop (bottom right) panels, reflecting that Rh has essentially no magnetic moment in the AFM phase [64]. In the FM (top right) panel, Rh may acquire an induced moment [65]. The vector *m* denotes the local Fe moment direction, and B (red) is the applied magnetic field. The AFM, FM, and spin‐flop configurations illustrate the possible field‐induced spin states relevant to Berry curvature generation. (b) Temperature dependence of anomalous Hall resistivity ρ_AHE_​ (left axis, red triangles) and magnetization M (right axis, blue triangles). While ρ_AHE_​ increases with decreasing temperature, M remains nearly constant from 150 to 5 K, yielding a moderate correlation coefficient r ∼0.697. This decoupling suggests that the AHE does not arise from remanent magnetization. (c) Scaling plot of ρ_AHE_​ vs. $\rho_{xx}^{2}$​, fitted with the intrinsic AHE expression $\rho_{\mathrm{AHE}}=f\left(c\right)+\sigma_{xy}^{int}\rho_{xx}^{2}$​. The linear dependence and the extracted intrinsic Hall conductivity $\sigma_{xy}^{int}$∼116 Ω^−1^cm^−1^ confirms that the observed AHE originates from Berry curvature depletion, induced by spin canting in the antiferromagnetic phase.

The anomalous Hall effect in ferromagnets and in certain antiferromagnets arises in phases that break time‐reversal symmetry, where the combination of magnetic order and spin–orbit coupling produces a net Berry curvature. Consequently, this effect is typically absent in bulk collinear AFMs, whose net magnetization vanishes owing to exact compensation and the presence of combined inversion (*P*) and time‐reversal (*T*) symmetry (*PT* symmetry). Interestingly, recent theoretical and experimental studies have challenged this conventional viewpoint. Figure 3a schematically illustrates the microscopic mechanism underlying this phenomenon in our (110)‐oriented FeRh system. In conventional ferromagnetic order (top‐right in Figure 3a), magnetic moments align parallel, generating a finite net magnetization. Conversely, in an ideal antiferromagnetic state (bottom‐left in Figure 3a, $\vec{B}$//$\vec{m}$, where vector *m* denotes the local Fe moment direction), the moments are arranged in an antiparallel manner, cancelling each other out completely, thus eliminating net magnetization. Crucially, in the case of (110)‐oriented FeRh grown epitaxially on Al_2_O_3_ substrates, the crystal symmetry and atomic arrangement differ significantly from those of conventional FeRh films (typically oriented along (001)). Specifically, Fe and Rh atoms share the same atomic plane along the (110) crystallographic orientation (bottom‐right in Figure 3a, $\vec{B}⊥\vec{m}$), unlike their spatially separated arrangement in (001)‐oriented structures. This distinctive atomic plane configuration is particularly susceptible to spin reorientation when subjected to substrate‐induced interfacial strain and external magnetic fields. Under certain applied magnetic fields oriented parallel to the atomic plane, exchange interactions between Fe spins and neighboring nonmagnetic Rh atoms induce a subtle spin canting, known as spin‐flop coupling (bottom‐right panel). Unlike (001)‐oriented films, the (110) geometry on Al_2_O_3_ breaks spatial inversion symmetry due to the epitaxial stacking and interface strain, thereby lifting the *PT* constraint. Our non‐collinear DFT calculations indicate that the combined effect of (110) orientation and strain induces a subtle spin‐flop or canted alignment (see Figure S5). Although the resulting configuration remains fundamentally antiferromagnetic, this symmetry lowering enables a non‐vanishing Berry curvature in momentum space, acting as an effective magnetic field that generates V_AHE_ without requiring large macroscopic magnetization.

To clarify the underlying mechanism responsible for the AHE observed in our (110)‐oriented FeRh thin films, we systematically examined the temperature dependence of the anomalous Hall resistivity (ρ_AHE_​) in comparison with that of the measured magnetization (M) (Figure 3b). Conventionally, if the observed AHE were predominantly driven by extrinsic effects associated with remanent magnetization or uncompensated spins, one would anticipate a close correlation between ρ_AHE_​ and M as a function of temperature. However, our experimental results show that the M remains essentially constant (∼150 emu/cm^3^, representing a residual ferromagnetic fraction of approximately 10 %–15 %) across the temperature range of 5–150 K, exhibiting only minimal changes and without a substantial increase at low temperatures. In stark contrast, ρ_AHE_​​ significantly increases upon cooling, progressively reaching its highest magnitude at the lowest temperature. A quantitative analysis of these datasets reveals only a moderate correlation (correlation coefficient (r) of approximately 0.69), underscoring that changes in the magnetization alone cannot fully account for the observed anomalous Hall response. Such a moderate correlation strongly suggests that the underlying physics is dominated by intrinsic mechanisms rather than by extrinsic factors linked to residual magnetic moments or surface‐induced uncompensated spins.

Motivated by these results, we further explored whether the intrinsic nature of the anomalous Hall response in the FeRh films arises from the nonzero Berry curvature induced by subtle symmetry breaking, particularly the breaking of the time reversal symmetry associated with spin canting or spin flop coupling in the collinear antiferromagnetic state. To verify this scenario, we investigated in detail the scaling relationship between the AHC (σ_AHE_) and the longitudinal resistivity (ρ_xx_​) of these films (Figure 3c). Theoretical studies have shown that the AHC can be expressed as follows: $\sigma_{\mathrm{AHE}}=\rho_{\mathrm{AHE}}\;/\left(\rho_{xy}^{2}+\rho_{xx}^{2}\right)$, where the ρ_xy_ refers to Hall resistivity. For further quantitative analysis, we expressed the anomalous Hall resistivity explicitly as $\rho_{\mathrm{AHE}}=f\left(c\right)+\sigma_{xy}^{int}\rho_{xx}^{2}$, where *f*(*c*) represents the constant offset arising from extrinsic contributions and $\sigma_{xy}^{int}$​ denotes the intrinsic AHC directly connected to the Berry curvature of the system. Because ρ_xx_ was easily obtained using a standard four‐probe measurement (see Figure S2), we plotted ρ_AHE_​ as a function of $\rho_{xx}^{2}$​, revealing a clear linear relationship (Figure 3c). This linear scaling behavior is characteristic of the intrinsic anomalous Hall regime, in which the slope directly reflects the contribution of the Berry curvature. Physically, the intrinsic regime arises from the Berry curvature acting as an effective magnetic field in momentum space, deflecting charge carriers according to the semiclassical equation of motion:

(1) $$\frac{d\langle \vec{r}\rangle }{dt}=\frac{\partial E}{h\partial \vec{k}}+\frac{e}{h}E\times \Omega_{k}$$

Here, Ω_*k*_​ represents the Berry curvature distribution in momentum space. A linear fit of our data across the temperature range from 5 to 100 K yields an $\sigma_{xy}^{int}$ of approximately 116 Ω^−1^ cm^−1^. This substantial intrinsic conductivity strongly suggests a prominent Berry curvature effect independent of variations in the magnetization.

Taken together, the magnetization‐insensitive behavior of the ρ_AHE_ and the quadratic scaling between σ_AHE_ and $\rho_{xx}^{2}$ provide compelling evidence that the observed AHE originates from an intrinsic mechanism driven by the Berry curvature, rather than from extrinsic sources such as residual magnetization. Furthermore, the ‘unusual’ non‐saturating profile of the V_AHE_ vs. magnetic field curves (Figure 2e) is a characteristic feature of this Berry curvature‐driven response. Unlike conventional ferromagnets, where the AHE saturates with magnetization, the continuous increase in V_AHE_ reflects the progressive redistribution of the Berry curvature in the Brillouin zone as the spin canting angle evolves with the external field, as supported by our theoretical calculations (Figure S5). This interpretation is further supported by recent theoretical and experimental work, including the study by Bera et al. [57], where a similar scaling relation was employed to identify Berry curvature‐driven transport in symmetry‐broken systems. In this context, the linear relation between σ_AHE_ and $\rho_{xx}^{2}$, observed in the (110)‐oriented FeRh films in this study, is consistent with the Karplus–Luttinger‐type intrinsic AHE mechanism, wherein the slope reflects the strength of the Berry curvature. These findings reinforce the view that crystallographic orientation and strain‐induced symmetry breaking can activate topological transport phenomena even in collinear AFMs, enabling finite AHE signals without net magnetization.

### 2.4 Large Anomalous Hall Factor With Modest Hall Angle in (110)‐Oriented FeRh Films

**FIGURE 4:**
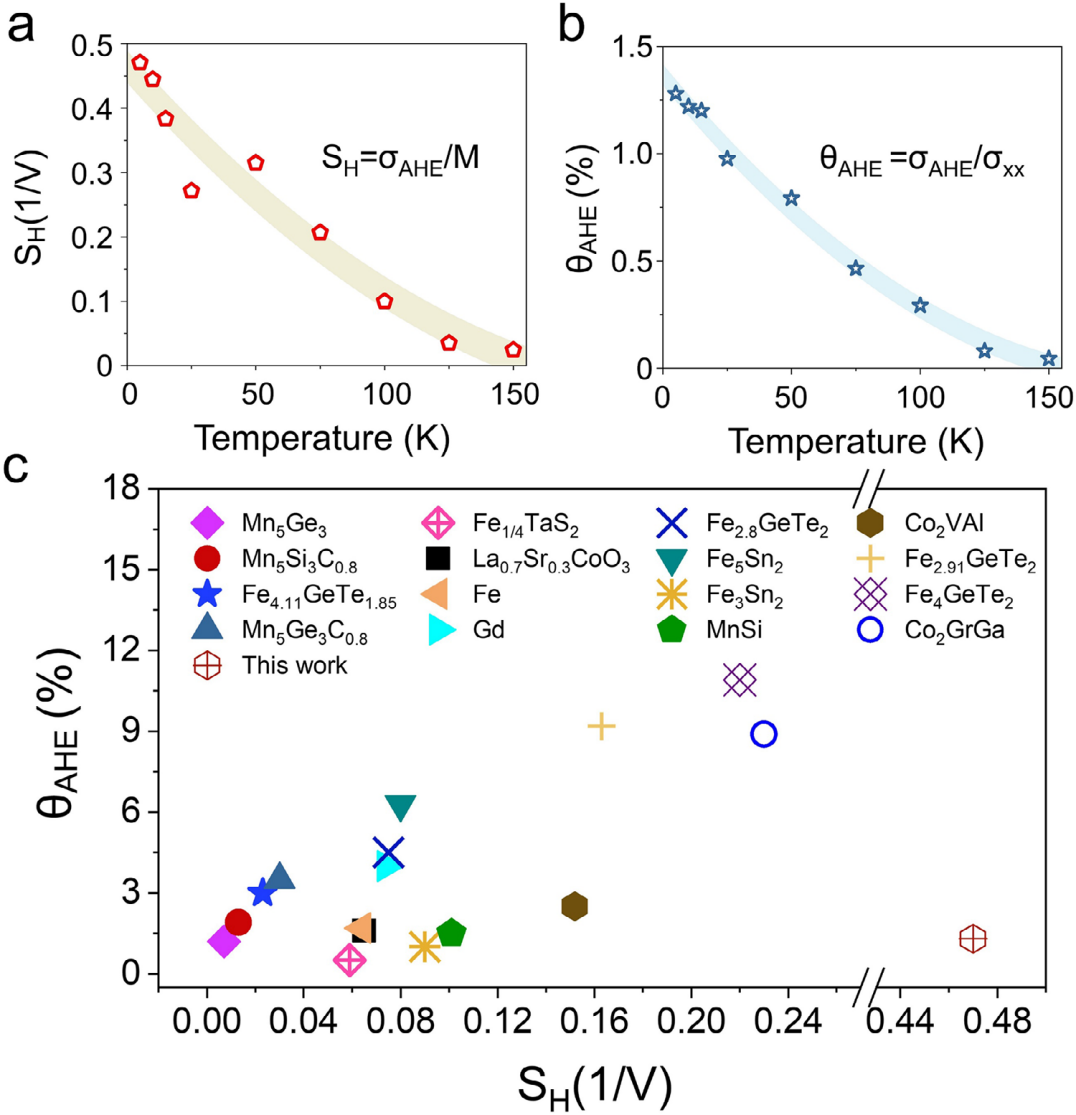
Anomalous Hall factor and anomalous Hall angle of (110)‐oriented FeRh thin films. (a) Temperature dependence of the anomalous Hall factor S_H_ = σ_AHE_/M, representing the transverse Hall response normalized by the magnetization. The value of S_H_ increases sharply at lower temperatures, indicating an enhanced anomalous Hall response per unit magnetization. (b) Temperature dependence of the anomalous Hall angle θ_AHE_ = σ_AHE_/σ_xx_​, which quantifies the transverse charge deflection efficiency. The angle decreases monotonically as temperature increases, reflecting the suppression of the Berry curvature effect at higher temperatures. (c) Comparison of the anomalous Hall factor and angle in this study (red hexagon) with various reported magnetic systems, including FMs and topological materials. Notably, the data from this study exhibit a relatively large anomalous Hall factor despite a moderate Hall angle, suggesting a significant intrinsic contribution from the Berry curvature and minimal extrinsic scattering effects.

Building upon these findings, we further evaluated the intrinsic anomalous Hall response using two key transport parameters: the anomalous Hall factor (S_H_​) and anomalous Hall angle (θ_AHE_). The S_H_, defined as the σ_AHE_ divided by M, provides insight into the efficiency with which the intrinsic Berry curvature generates a transverse voltage per unit of magnetization. Simultaneously, the θ_AHE_, defined as the ratio of σ_AHE_ to longitudinal conductivity (σ_xx_​), quantifies the efficiency of transverse charge deflection relative to the longitudinal charge conduction. Figure 4a,b displays the temperature‐dependent behaviors of S_H_ ​and θ_AHE_, measured in the (110)‐oriented FeRh films, respectively. While both parameters decrease monotonically with increasing temperature, the absolute magnitude of S_H_ remains significantly large even as the θ_AHE_ becomes relatively modest. To highlight the significance of this result, we compared our data with those of a broad range of magnetic materials previously reported, including metallic FMs (Figure 4c) [57]. Remarkably, the (110)‐oriented FeRh films exhibit an exceptionally large anomalous Hall factor (S_H_ ∼ 0.46 V^−1^ at 5 K), placing it clearly at the upper bound of reported values. However, the corresponding anomalous Hall angle is comparatively small (θ_AHE_ ∼ 1.3 % at 5 K). This combination, a large S_H_ coupled with a relatively small θ_AHE_, indicates a pronounced intrinsic Berry curvature contribution and minimal extrinsic influences, such as scattering from residual magnetization or structural imperfections. Practically, this combination indicates that substantial intrinsic Berry curvature can generate a measurable anomalous Hall voltage even when the overall transverse charge deflection efficiency, reflected by the anomalous Hall angle, remains moderate.

**FIGURE 5:**
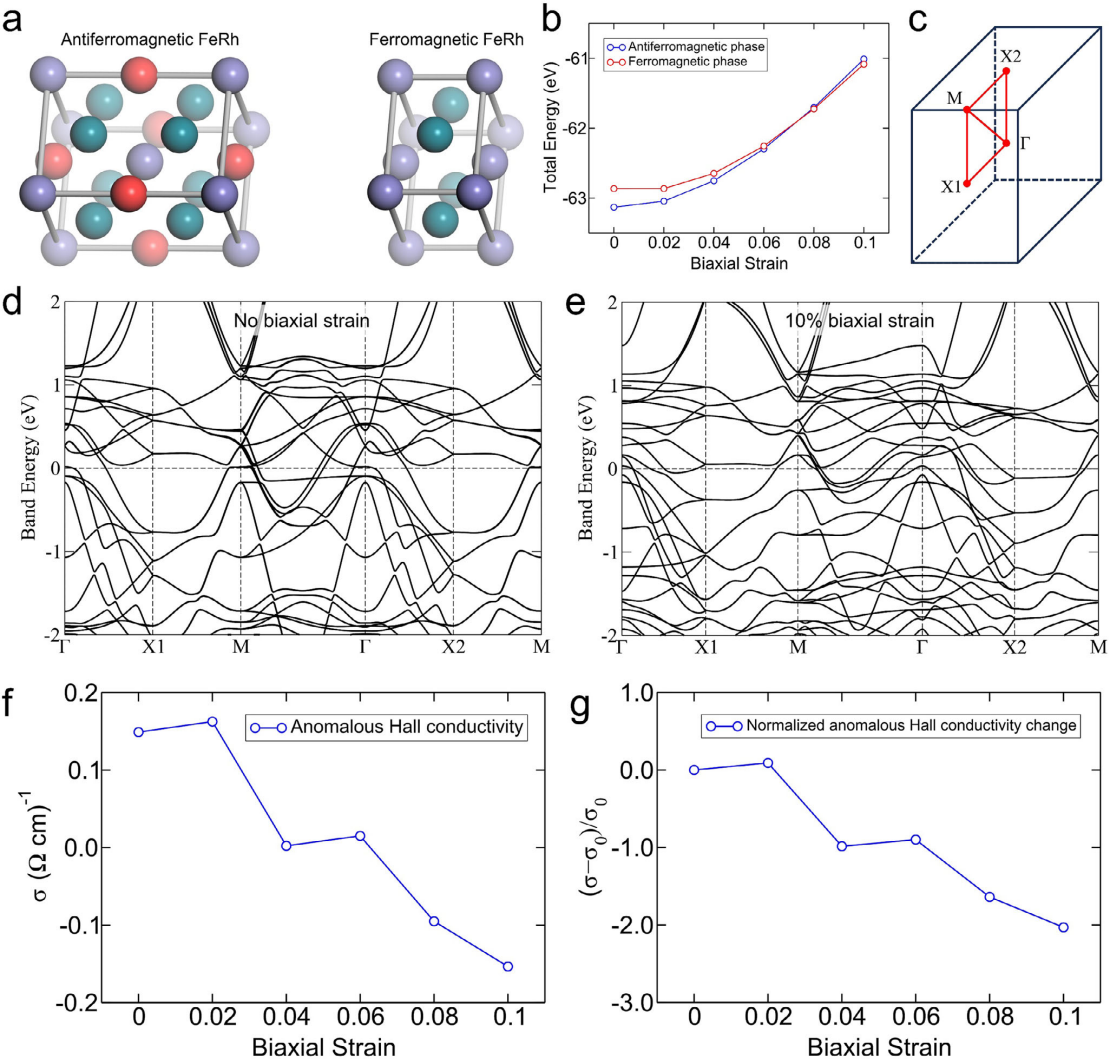
First‐principles analysis of strain‐induced modulation of electronic structure and anomalous Hall conductivity in antiferromagnetic FeRh. (a) Ball‐and‐stick model of a unit cell of antiferromagnetic FeRh. Purple(red) balls stand for Fe atoms with spin up(down), and turquoise balls stand for Rh atoms. Biaxial tensile strains are applied in a (110) plane, which corresponds to the top plane of each unit cell. (b) Total energies of the antiferromagnetic phase and the ferromagnetic phase of FeRh depending on biaxial strain along in‐plane directions. The total energies per 8 Fe atoms and 8 Rh atoms are shown. (c) First Brillouin zone of the antiferromagnetic phase of FeRh with no biaxial strain. The Γ‐X1 and Γ‐X2 lines are oriented along mutually perpendicular (110) directions. The biaxial strain is applied in a plane perpendicular to the Γ‐X2 direction. Electronic band structure of the antiferromagnetic phase of FeRh with (d) no and (e) 10 % biaxial strain. (f) Anomalous Hall conductivities of the antiferromagnetic phase of FeRh depending on biaxial strain along in‐plane directions. (g) Normalized anomalous Hall conductivities of the antiferromagnetic phase of FeRh depending on biaxial strain along in‐plane directions.

To support our experimental data, we calculated the total energies, electronic structures, and the anomalous Hall conductivities of FeRh structures with and without the tensile biaxial strains along the plane (Figures 5a–g; S6–S9). We clarify that the primary objective of these first‐principles calculations is to identify strain‐induced trends in the electronic structure that rationalize our experimental observations, rather than to provide a quantitatively exact description of correlated electronic states. The total energies of the antiferromagnetic and ferromagnetic phases of FeRh are shown in Figure 5b, indicating that the antiferromagnetic phase of FeRh is favored in moderate tensile strains. We estimate that the ferromagnetic phase of FeRh will appear with the application of a strain of approximately 0.06–0.08. It is important to note that this value refers exclusively to the theoretical strain scale investigated in our calculations to identify the energetic boundaries between the AFM and FM phases, where a larger strain scale is required to resolve clear trends within numerical uncertainties. This does not represent the actual epitaxial strain realized in our experiments, which, as evidenced by the structural analysis in Figure 1, remains at the sub‐percent to few‐percent level and is partially relaxed across the film thickness. With no strain, the shape of the first Brillouin zone is rectangular with two edges equal in length. Therefore, the lengths of the Γ‐X1 line and the Γ‐X2 line are equal, as shown in Figure 5c. With the application of the biaxial tensile strain in the plane perpendicular to the Γ‐X2 direction, the length of the Γ‐X1 line decreases, whereas that of the Γ‐X2 line remains the same. The calculated electronic structures are shown in Figure 5d,e. The Fermi levels are set to 0, and the horizontal dashed lines indicate the Fermi levels. The electronic structures have different features along the Γ‐X1 and Γ‐X2 lines, even under no strain. For example, the conduction bands of antiferromagnetic FeRh have different band crossing points. This is not surprising because the major components of the spin magnetic moment of the Fe atoms are parallel to the Γ‐X2 direction, which breaks the rotational symmetry. We note that the feature along the Γ‐X1 line rather than along the Γ‐X2 line in Figure 5d is very close to the previous calculation in the literature [58]. The different features become more pronounced with the increasing magnitude of the tensile strain. It is remarkable that the conduction band just above the Fermi level along the X1‐M line is almost flat toward the X1 point, and the region approaches the Fermi level as the biaxial strain increases, as shown in Figures 5d,e, and S5. Moreover, the shape of the Fermi surface changes significantly, as can be inferred from the position of the crossing points of the electronic structures at the Fermi level. We believe that the sensitive dependence of the shape of the conduction bands on the biaxial strain can be utilized to fine‐tune the electrical properties of the (110) FeRh antiferromagnetic films. The AHCs of the antiferromagnetic phase of FeRh are shown in Figure 5f, g. Overall, with the application of the tensile biaxial strains, the conductivities show a decreasing trend. This trend is attributed to the systematic change in electronic structure with the strains, as explained in the sharp difference between the features along the Γ‐X1 line and those along the Γ‐X2 line. The calculated results are consistent with the experimental measurements at the level of strain‐driven physical trends. While a direct quantitative comparison of absolute magnitudes is constrained by finite‐temperature effects and impurity scattering in the experiments, both theory and experiment consistently demonstrate that symmetry‐lowering in the (110) geometry activates the intrinsic Berry‐curvature‐driven transport in the antiferromagnetic regime.

These observations suggest that (110)‐oriented FeRh films offer a distinctive platform for antiferromagnetic spintronic devices. Their substantial anomalous Hall factor enables robust intrinsic Hall responses without relying on large magnetization or extrinsic scattering. Consequently, this approach provides a pathway toward electrically readable antiferromagnetic spintronic applications, where intrinsic Berry curvature‐driven effects can be precisely engineered through crystallographic orientation and strain.

## 3 Conclusion

In summary, we demonstrate that epitaxially grown (110) oriented FeRh thin films on Al_2_O_3_ substrates exhibit a pronounced anomalous Hall effect, while retaining an antiferromagnetic‐dominant state throughout the range of temperatures from 5 to 300 K. Unlike the bulk B2 FeRh phase where the combined inversion (*P*) and time‐reversal (*T*) symmetry (*PT* symmetry) enforces a vanishing Berry curvature, the epitaxial (110) orientation and substrate‐induced strain break the spatial inversion symmetry. This symmetry‐lowering mechanism lifts the *PT* constraint, thereby activating an intrinsic Berry curvature‐driven anomalous Hall response even in a collinear antiferromagnetic configuration. These findings provide important insights for the development of antiferromagnetic spintronics.

The ability to electrically detect the Hall signal in a system with negligible net magnetization, originating from an intrinsic topological mechanism rather than extrinsic ferromagnetic residues, offers a promising means of probing and controlling antiferromagnetic order. FeRh is particularly attractive for applications due to its magnetic phase transition at room temperature and its compatibility with layered device architectures. Future memory or sensor devices could store information in the antiferromagnetic state and enable readout through the anomalous Hall voltage, combining the robustness and speed of AFMs with straightforward electrical detection. More broadly, our results highlight how crystallographic orientation and strain‐induced symmetry breaking can activate Berry curvature‐driven transport in a wide range of antiferromagnetic materials. By establishing (110)‐oriented FeRh as a symmetry‐tunable metamagnetic platform, this work advances the fundamental understanding of Berry curvature in AFMs and paves the way for practical antiferromagnetic spintronic technologies.

## 4 Experimental Section

### 4.1 Film Growth and Characterization

(110)‐oriented FeRh thin films with a nominal thickness of ∼25 nm were deposited on single‐crystalline Al_2_O_3_ substrates using DC magnetron sputtering. Before deposition, the substrates were cleaned in an argon ambient (1 × 10^−2^ Torr) using radio‐frequency plasma (10 W) to remove organic contaminants. The FeRh layer was grown from a stoichiometric FeRh sintered 2‐inch target under a working argon pressure of 4 × 10^−3^ Torr and a DC power of 75 W. The deposition was carried out at a substrate temperature of 630°C with a growth rate of approximately 0.04 nm s^−1^. Post‐deposition, the films were annealed at 730°C for 1 h in a high‐vacuum environment (∼4 × 10^−8^ Torr) to enhance crystallinity and chemical ordering. Structural and phase characterizations of the films were performed using HR‐XRD, cross‐sectional high‐resolution transmission electron microscopy (HR‐TEM), and STEM.

### 4.2 Magnetization Measurements

Magnetization measurements were performed using a VSM integrated into a Quantum Design PPMS (Dynacool), operating over a temperature range of 100–350 K and under magnetic fields of up to 9 T. The external magnetic field was applied in the plane of the sample. Data were acquired during both warming and cooling cycles to probe the thermal hysteresis associated with the magnetic phase transition.

### 4.3 Hall Effect and Electrical Transport Measurements

The electrical resistivity and AHE were measured on FeRh films (∼25 nm thick) patterned into a Hall bar geometry using standard photolithography and ion milling techniques. A constant current of 5 µA was applied along the longitudinal direction of the device. To eliminate spurious contributions from contact misalignment or geometric asymmetry, the transverse (Hall) voltages were recorded under both positive and negative magnetic field polarities, and the antisymmetric component was extracted by averaging them. Longitudinal resistivity was measured simultaneously using a standard four‐probe method. Temperature‐dependent measurements of AHE and magnetoresistance were carried out over the range of 5–300 K.

### 4.4 First Principles Calculations for FeRh

The calculations were performed using the ab‐initio total‐energy and molecular‐dynamics Vienna ab‐initio simulation program (VASP) developed at the Institut für Materialphysik of the Universität Wien, using the projector‐augmented‐wave (PAW) approach [59] and Perdew–Burke–Ernzerhof (PBE) generalized gradient approximation [60]. A cutoff energy of 520 eV was used for the plane‐wave basis set. For sampling the Brillouin zone, the Monkhorst–Pack scheme was used. The k‐point meshes for self‐consistent charge density were 12 × 12 × 8 for antiferromagnetic FeRh and 12 × 12 × 16 for ferromagnetic FeRh. The unit cells are shown in Figure 5a. Biaxial strains up to 10 % along in‐plane directions were applied. The wavefunctions obtained from the self‐consistent calculations were projected on 12 maximally‐localized Wannier functions (MLWFs) per atom using the WANNIER90 code [61]. The AHCs were calculated by integrating the Berry curvature over the occupied states in the Brillouin zone using a 96 × 96 × 64 k‐point mesh for antiferromagnetic FeRh and a 96 × 96 × 128 k‐point mesh for ferromagnetic FeRh with adaptive refinement.

To further examine the stability of non‐collinear magnetic configurations under strain and spin–orbit coupling, complementary first‐principles calculations were performed using the OpenMX code. These calculations were focused on the evolution of magnetic configurations and spin canting, rather than on electronic transport properties. Non‐collinear density functional theory calculations were performed using the OpenMX [62, 63] code based on the linear combination of pseudo‐atomic orbitals (LCPAO) method. The exchange–correlation interaction was treated within the Perdew–Burke–Ernzerhof (PBE) generalized gradient approximation. Spin–orbit coupling (SOC) was included self‐consistently using fully relativistic, j‐dependent norm‐conserving pseudopotentials. The electronic wavefunctions were expanded using optimized pseudo‐atomic basis sets of Fe5.0S‐s2p2d2f1 and Rh7.0‐s2p2d2f1. Biaxial strain was systematically applied with values of 0.00, 0.02, 0.04, 0.06, 0.08, and 0.10, defined relative to the unstrained lattice parameters, and both (100)‐ and (110)‐oriented structures were considered for each strain value. All calculations were initialized with a G‐type antiferromagnetic (AFM‐G) configuration, and the spin degrees of freedom were allowed to relax fully within a non‐collinear magnetic framework while keeping the underlying lattice structure fixed. The Brillouin zone was sampled using Monkhorst–Pack k‐point meshes chosen to maintain a uniform k‐point density corresponding to a real‐space length of approximately 40 Å, ensuring consistent sampling accuracy across all strained systems. A real‐space energy cutoff of 500 Ry was employed for numerical grid integration.

## Author Contributions

The manuscript was written through the contributions of all authors. All authors have approved the final version of the manuscript. § These authors contributed equally. Sang‐Kwon Lee conceived and designed the study. Yun‐Ho Kim, Jae Won Choi, and Jung‐Min Cho conducted the AHE measurements using the PPMS setup. Shelby Fields and Steven Bennett fabricated the FeRh thin films for the AHE measurements. Gil‐Sung Kim conducted HR‐XRD, HR‐TEM, and STEM. Yeonho Choi, Hongkee Yoon, and Young‐Gui Yoon conducted the first‐principles calculation of ferromagnetic and antiferromagnetic FeRh structures. Yun‐Ho Kim, Gil‐Sung Kim, Won‐Yong Lee, and Sang‐Kwon Lee analyzed the AHE characterization. Gil‐Sung Kim, Won‐Yong Lee, Mona Zebarjadi, Steven Bennett, and Sang‐Kwon Lee supervised the experiments. Yun‐Ho Kim, Gil‐Sung Kim, Won‐Yong Lee, Young‐Gui Yoon, and Sang‐Kwon Lee wrote the manuscript. All the authors discussed the results and commented on the manuscript.

## Conflicts of Interest

The authors declare no conflicts of interest.

## Supporting information




**Supporting File**: advs74119‐sup‐0001‐SuppMat.docx.

## Data Availability

The data that support the findings of this study are available from the corresponding author upon reasonable request.
