# Supplementary material for: Large Anomalous Hall Effect, Non‐Vanishing Berry Curvature in (110) FeRh Antiferromagnet Films via Interface Strain
Source: Adv Sci (Weinh). 2026 Jan 28;13(19):e18999. doi: 10.1002/advs.202518999 (PMC13045218; doi:10.1002/advs.202518999)
Supplement: Supplementary file 1 — Supporting File: advs74119‐sup‐0001‐SuppMat.docx. [file ADVS-13-e18999-s001.docx]

Supporting Information

Large Anomalous Hall Effect, Non-Vanishing Berry Curvature in (110) FeRh Antiferromagnet Films via Interface Strain

Yun-Ho Kim ^a§^, Gil-Sung Kim ^a§^, Jae Won Choi ^a^, Jung-Min Cho ^a^, Won-Yong Lee ^b^, Hongkee Yoon ^b^, Yeonho Choi ^a^, Shelby Fields ^c^, Steven Bennett ^c^, Mona Zebarjadi ^d,e^, Young-Gui Yoon ^a^ and Sang-Kwon Lee ^a*^

^a^ Department of Physics, Chung-Ang University, Seoul 06974, Republic of Korea

^b^ Department of Semiconductor Physics, Kangwon National University, Chuncheon 24341, Republic of Korea

^c^ Materials Science and Technology Division, U.S. Naval Research Laboratory, Washington, DC 20375, USA

^d^ Department of Electrical and Computer Engineering, University of Virginia, Charlottesville, VA 22904, USA

^e^ Department of Materials Science and Engineering, University of Virginia, Charlottesville, VA 22904, USA

^§^ These authors contributed equally to this work

^*^Address correspondence to [sangkwonlee@cau.ac.kr](mailto:sangkwonlee@cau.ac.kr)

**The PDF file includes:**

Supplementary note S1

Figs. S1 to S9

**Supplementary note S1. Decomposition and analysis of the Hall resistivity**

The measured Hall resistivity $\rho_{xy}$in magnetic conductors generally consists of the ordinary Hall effect (OHE), originating from the Lorentz force acting on charge carriers, and the anomalous Hall effect (AHE), which arises from spin–orbit coupling in the presence of magnetic order. Accordingly, the total Hall resistivity can be written as $\rho_{xy}(B)=R_{0}B+\rho_{xy}^{\mathrm{AHE}}$, where $R_{0}$is the ordinary Hall coefficient and $B$is the applied magnetic field, following the standard phenomenology of the anomalous Hall effect. To eliminate spurious contributions from longitudinal resistivity mixing due to contact misalignment, the raw transverse Hall signal was antisymmetrized with respect to the magnetic field as $\rho_{xy}(B)=[\rho_{xy}^{\mathrm{raw}}(+B)-\rho_{xy}^{\mathrm{raw}}(-B)]/2$, ensuring that only field-odd components were retained. The ordinary Hall coefficient $R_{0}$was determined by performing a linear fit to the high-field region of $\rho_{xy}(B)$, where the Hall resistivity exhibits a clear linear dependence on magnetic field and the anomalous Hall contribution is either saturated or varies weakly with field. The resulting linear term $R_{0}B$was then subtracted from the total Hall resistivity to obtain the anomalous Hall resistivity $\rho_{xy}^{\mathrm{AHE}}(B)$. To verify the robustness of this procedure, the fitting range within the linear high-field regime was varied, and the extracted values of $R_{0}$and $\rho_{xy}^{\mathrm{AHE}}$ were found to vary only within experimental uncertainty, without affecting the qualitative temperature and field dependence of the anomalous Hall signal. After removal of the ordinary Hall contribution, the anomalous Hall resistivity was further analyzed within the established scaling framework $\rho_{xy}^{\mathrm{AHE}}=a\rho_{xx}+b\rho_{xx}^{2}$, where the linear and quadratic terms are commonly associated with skew-scattering and intrinsic or side-jump mechanisms, respectively, providing a basis for discussing the origin of the anomalous Hall effect in the present system.

**Supplementary figures**


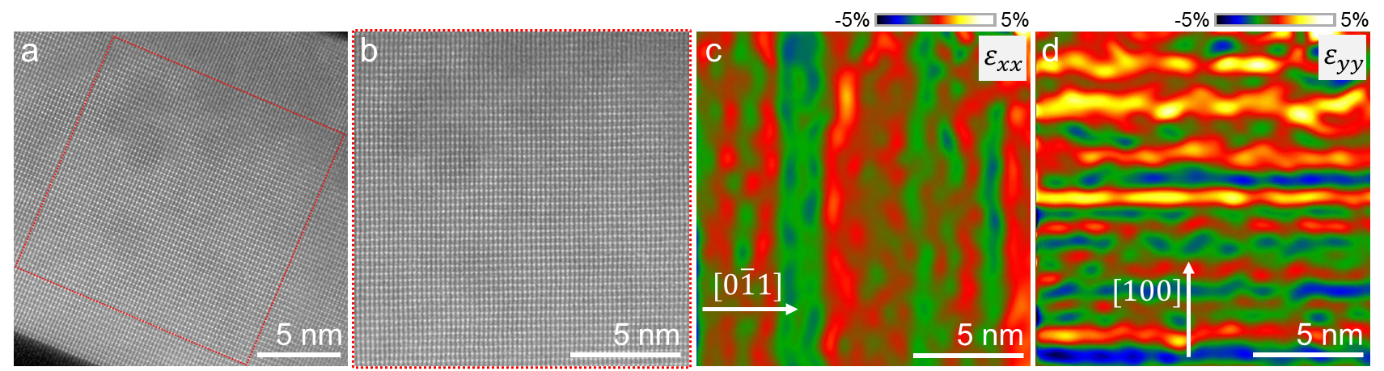


**Figure S1.** Lattice strain variation of the (110)-oriented FeRh thin film. (a) Low-magnification STEM image of the FeRh, where the GPA region is highlighted by a red dashed square. (b-d) Enlarged STEM image and corresponding in-plane ($\varepsilon_{xx}$) and out-of-plane strain ($\varepsilon_{yy}$) maps along [0$\bar{1}1$] and [$100$] directions.


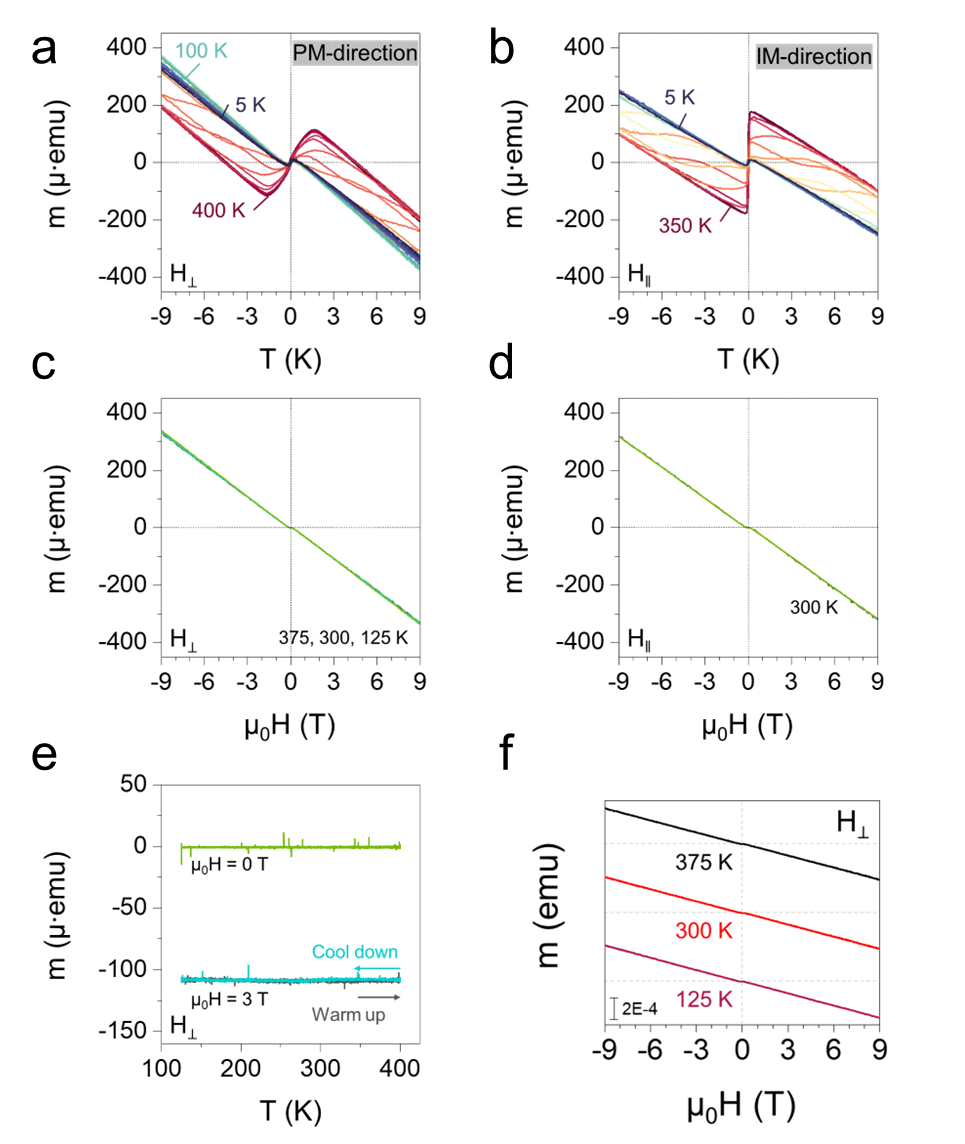


**Figure S2.** Temperature-dependent magnetization–field (M–H) curves measured by VSM. (a) M–H curves of a 25-nm-thick FeRh film grown on an Al_2_O_3_ (sapphire) substrate with the magnetic field applied perpendicular to the film plane (out-of-plane configuration). (b) M–H curves of the same FeRh (25 nm)/Al_2_O_3_ sample measured with the magnetic field applied parallel to the film plane (in-plane configuration). (c) Background VSM signal obtained from a bare Al_2_O_3_ substrate under an out-of-plane magnetic field, demonstrating the negligible magnetic contribution from the substrate. (d) M–H curve of the bare Al_2_O_3_ substrate measured with the magnetic field applied parallel to the substrate plane, confirming the absence of in-plane magnetic contribution. (e) Temperature-dependent magnetization (M–T) curve of the bare sapphire substrate, showing no appreciable change in magnetization over the measured temperature range. (f) Stacked M–H curves of the FeRh film at selected temperatures, highlighting the temperature evolution of the magnetic response.


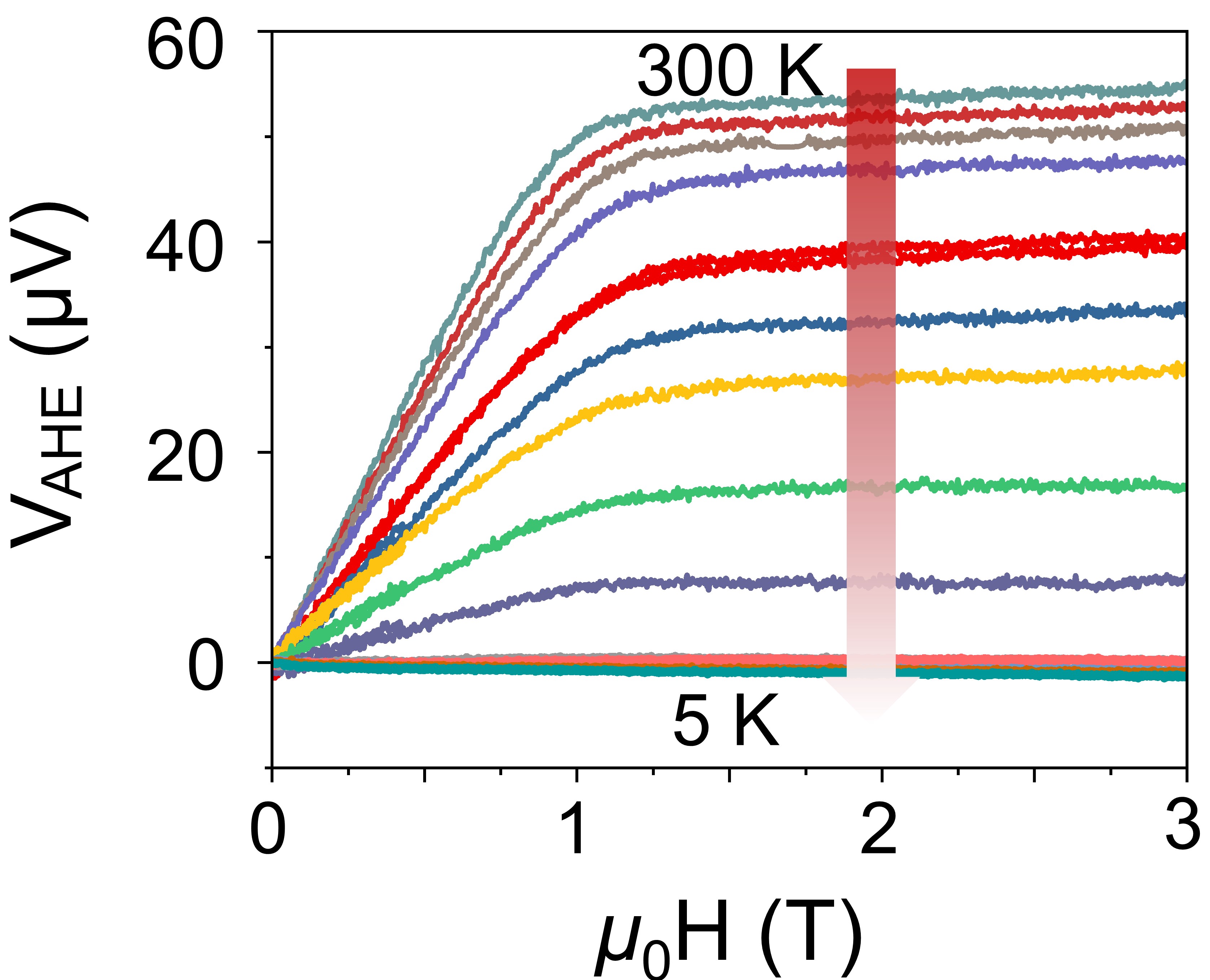


**Figure S3.** Temperature-dependent Hall measurement of FeRh thin film (80-nm-thick) on MgO substrate. Hall resistivity of an 80 nm-thick FeRh film deposited on a MgO substrate, measured from 300 K to 5 K. As the temperature decreases, the anomalous Hall effect (AHE) component gradually diminishes, indicating the suppression of ferromagnetic ordering and the progression toward the antiferromagnetic phase.


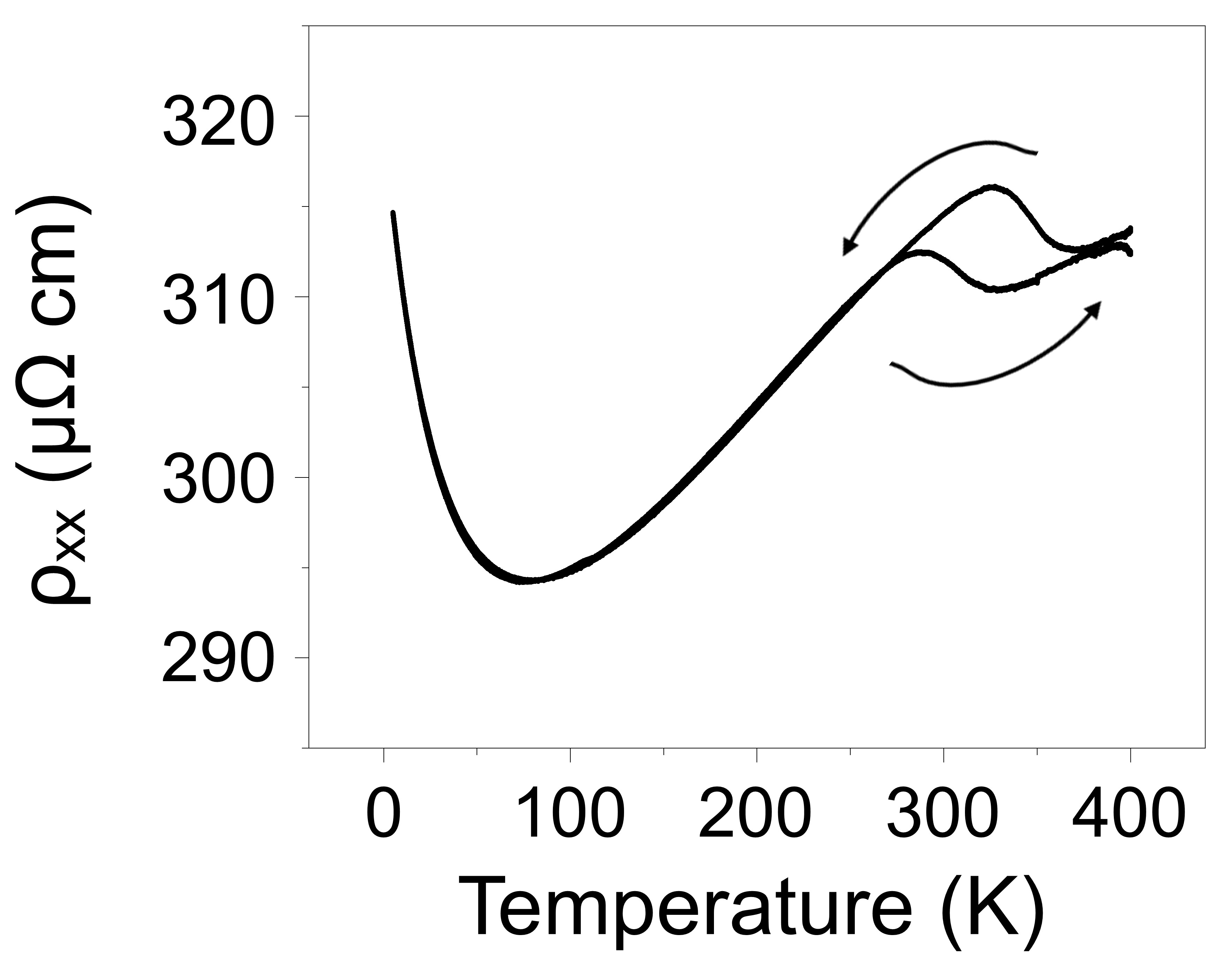


**Figure S4.** longitudinal resistivity ($\rho_{\mathrm{xx}}$​) of the (110)-oriented FeRh thin film (thickness of ~25 nm).


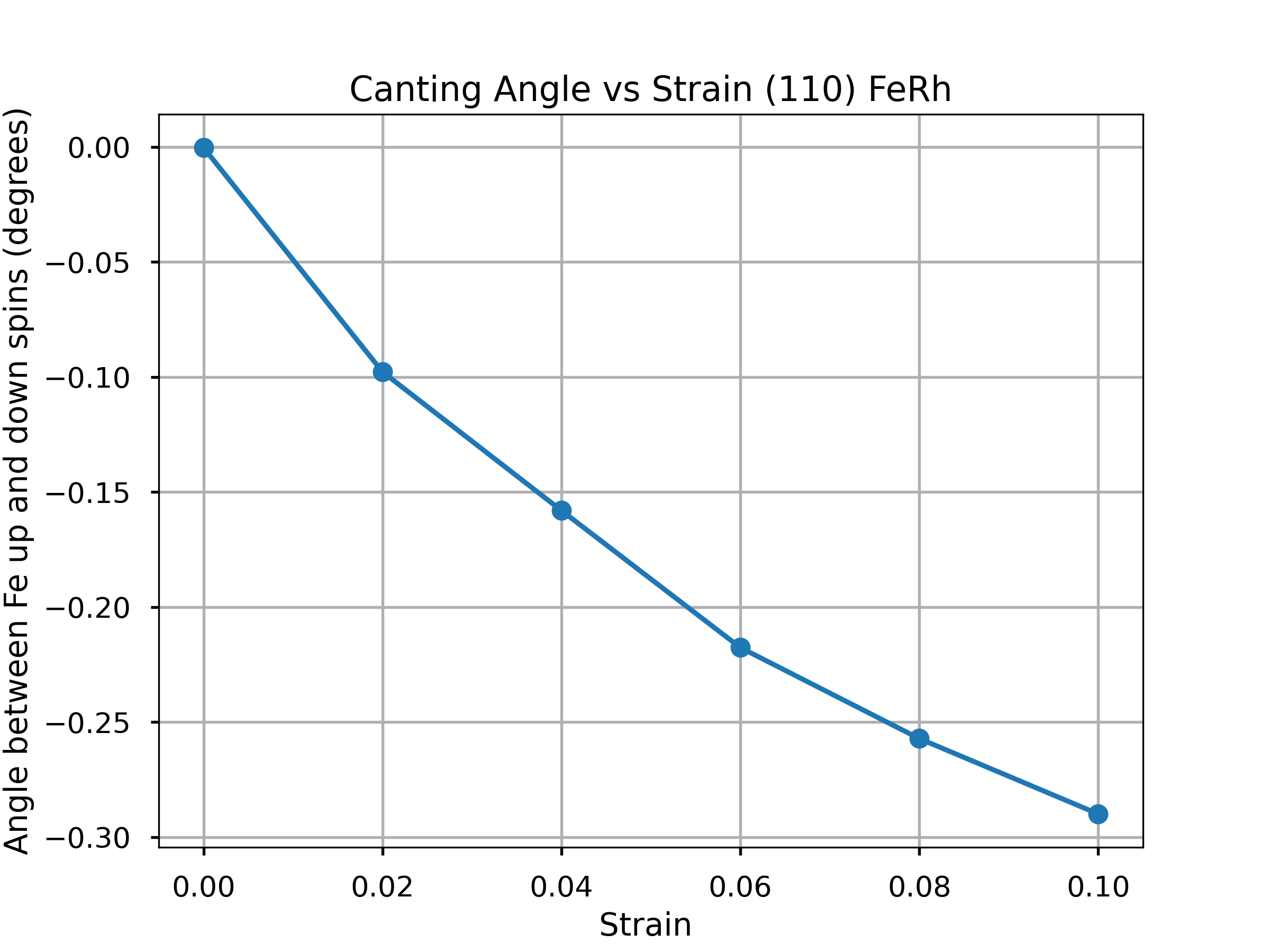


**Figure S5**. Strain dependence of the spin canting angle in (110)-oriented FeRh obtained from first-principles calculations. The canting angle is defined as the relative angle between the Fe moments on the two antiferromagnetic sublattices. For the unstrained structure, the system converges to a perfectly collinear antiferromagnetic ground state. A finite canting angle is observed only when tensile strain is applied along the out-of-plane direction, corresponding to a surface geometry with enhanced out-of-plane distortion, and when spin–orbit coupling is included. In contrast, for all other cases, including unstrained structures, strained configurations without the out-of-plane surface orientation, or calculations excluding spin–orbit coupling, the canting angle remains below 0.01°, indicating a robust collinear antiferromagnetic state. These results highlight that the combined effect of strain, surface orientation, and spin–orbit coupling is required to induce spin canting in FeRh.


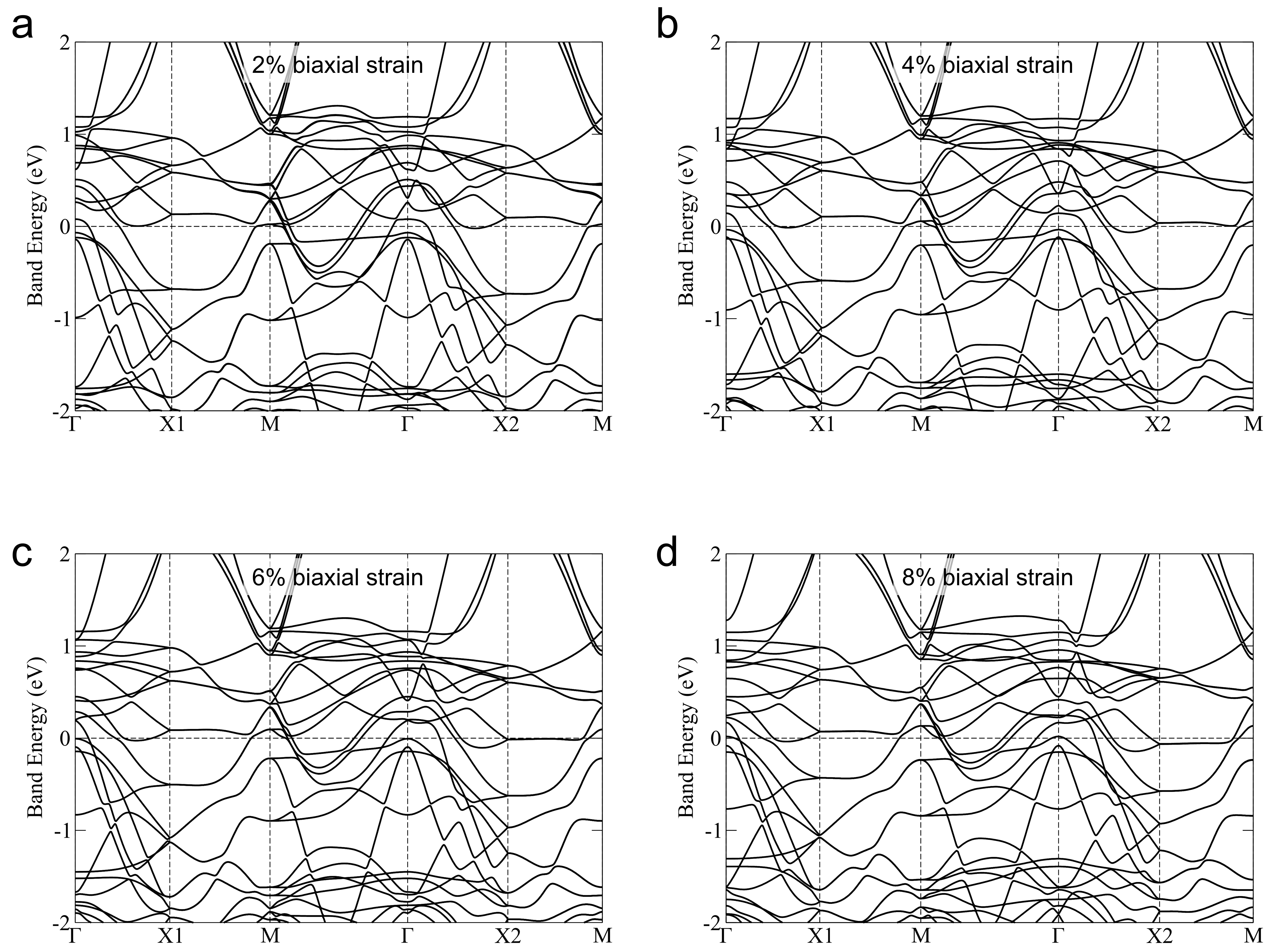


**Figure S6**. Electronic band structure of antiferromagnetic phase of FeRh with a) 2%, b) 4%, c) 6%, and d) 8% biaxial strain.





**Figure S7**. Electronic band structure of ferromagnetic phase of FeRh with a) no, b) 2%, c) 4%, d) 6%, e) 8%, and f) 10% biaxial strain.


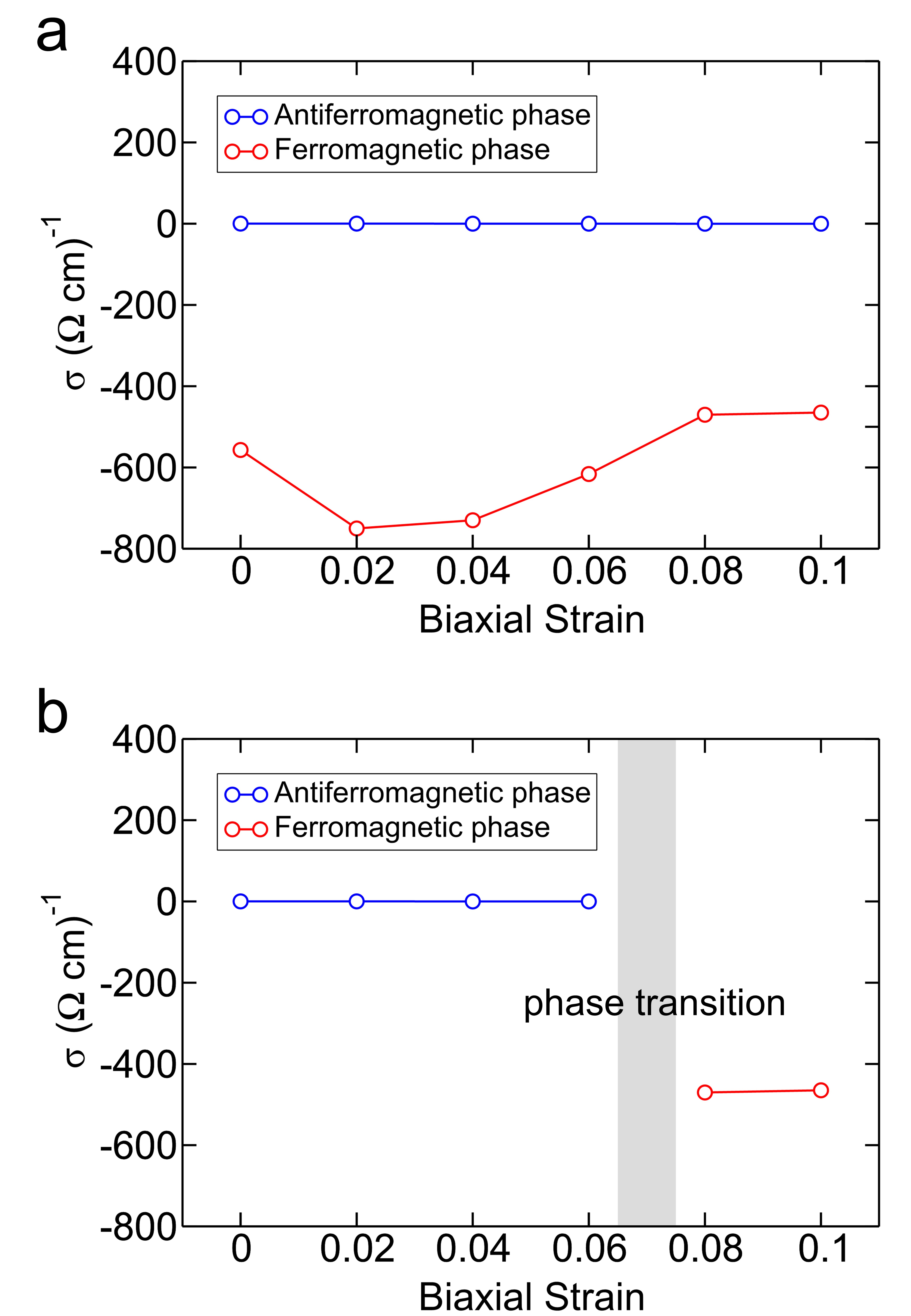


**Figure S8**. Anomalous Hall conductivities of antiferromagnetic phase and ferromagnetic phase of FeRh depending on biaxial strain along in-plane directions. Calculation results a) for each configuration and b) considering the phase transition under varying strain.


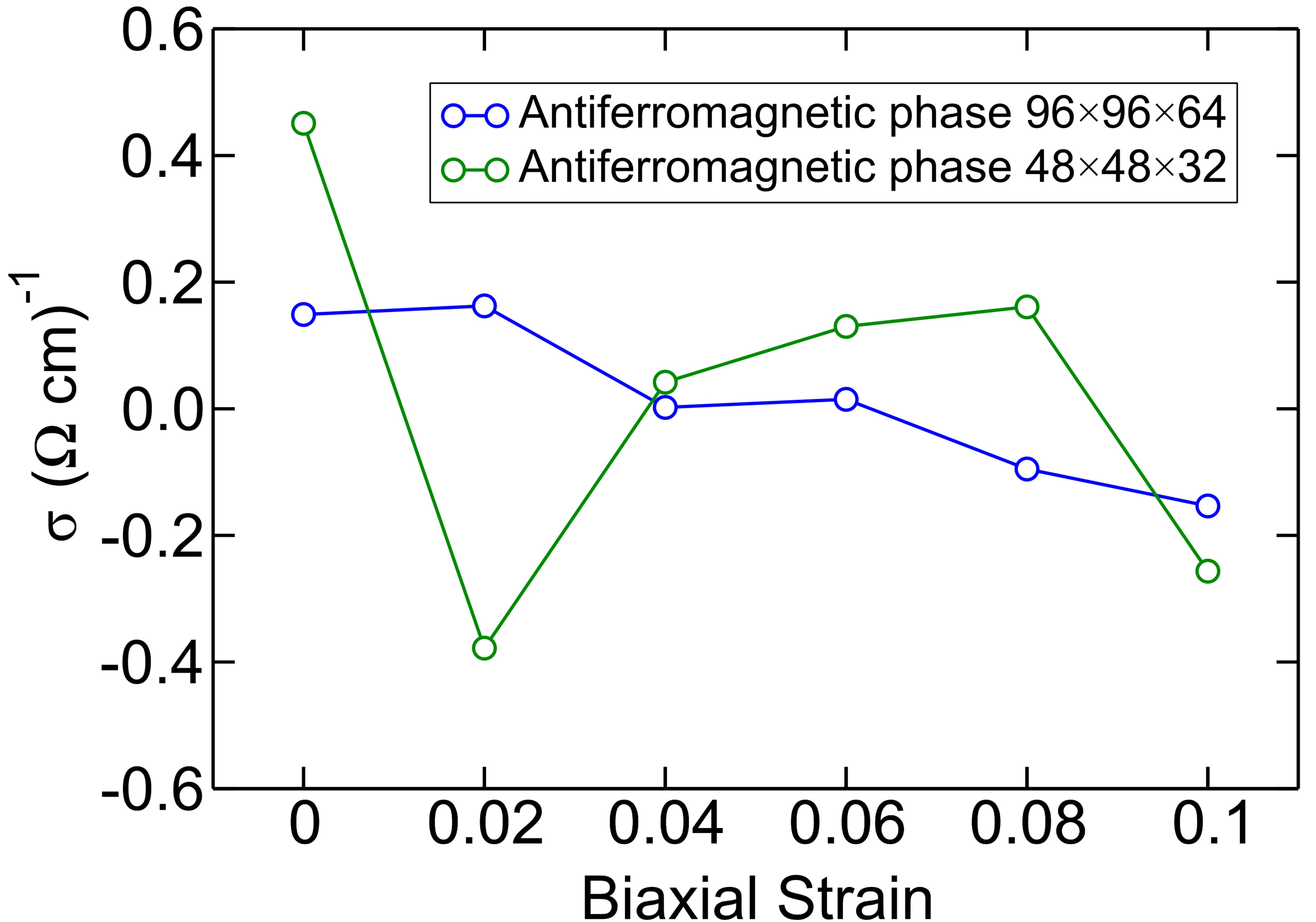


**Figure S9**. Calculated anomalous Hall conductivities of antiferromagnetic FeRh under biaxial strain with different k-point sampling densities. Two k-point grids (96×96×64 and 48×48×32) are compared to evaluate the convergence and resolution of Berry curvature sampling in the Brillouin zone.
